# *Mycobacterium tuberculosis* Type II Toxin-Antitoxin Systems: Genetic Polymorphisms and Functional Properties and the Possibility of Their Use for Genotyping

**DOI:** 10.1371/journal.pone.0143682

**Published:** 2015-12-14

**Authors:** Marina V. Zaychikova, Natalia V. Zakharevich, Maria O. Sagaidak, Nadezhda A. Bogolubova, Tatiana G. Smirnova, Sofya N. Andreevskaya, Elena E. Larionova, Maria G. Alekseeva, Larisa N. Chernousova, Valery N. Danilenko

**Affiliations:** 1 Vavilov Institute of General Genetics, Russian Academy of Sciences, Moscow, Russia; 2 Central Research Institute of Tuberculosis, Moscow, Russia; 3 Scientific Research Center for Biotechnology of Antibiotics "BIOAN", Moscow, Russia; 4 State University, Moscow Institute of Physics and Technology, Moscow, Russia; University of Waikato, NEW ZEALAND

## Abstract

Various genetic markers such as IS-elements, DR-elements, variable number tandem repeats (VNTR), single nucleotide polymorphisms (SNPs) in housekeeping genes and other groups of genes are being used for genotyping. We propose a different approach. We suggest the type II toxin-antitoxin (TA) systems, which play a significant role in the formation of pathogenicity, tolerance and persistence phenotypes, and thus in the survival of *Mycobacterium tuberculosis* in the host organism at various developmental stages (colonization, infection of macrophages, etc.), as the marker genes. Most genes of TA systems function together, forming a single network: an antitoxin from one pair may interact with toxins from other pairs and even from other families. In this work a bioinformatics analysis of genes of the type II TA systems from 173 sequenced genomes of *M*. *tuberculosis* was performed. A number of genes of type II TA systems were found to carry SNPs that correlate with specific genotypes. We propose a minimally sufficient set of genes of TA systems for separation of *M*. *tuberculosis* strains at nine basic genotype and for further division into subtypes. Using this set of genes, we genotyped a collection consisting of 62 clinical isolates of *M*. *tuberculosis*. The possibility of using our set of genes for genotyping using PCR is also demonstrated.

## Introduction

According to WHO data, tuberculosis (TB) is one of the deadliest among infectious and parasitic diseases with an estimate of 9 million new TB cases and 1.5 million deaths in 2013. Most of TB cases occur in African countries, Russia, Southeastern Asia and Middle East [http://www.who.int/tb/publications/global_report/en/]. Today the most dangerous forms of TB are not only those caused by drug-resistant strains of *Mycobacterium tuberculosis* [1], but also those with modified virulence, transmissibility and pathogenicity. A number of researches found a correlation between *M*. *tuberculosis* genotypes and their virulence and tendency to acquire drug resistance [2,3]. The *Mycobacteria* genus and strain identification is of great importance for the proper treatment assignment and epidemiologic situation evaluation.

Nowadays *M*. *tuberculosis* strains are classified in several genotypes, the basic of which are Beijing, X, Delhi/CAS, LAM, Haarlem, EAI, T, Ural and S [4,5]. Several genotyping techniques are available for *M*. *tuberculosis*, using different genetic markers [6].

Spoligotyping is based on polymorphism of the DR (direct repeat) locus on the chromosome, which contains a variable number of 36 base pairs (bp), separated by unique sequences (spacers) with length from 34 to 41 bp [7]. IS6110-RFLP-typing is based on the analysis of the restriction fragment length polymorphism obtained by the digestion of the genomic DNA at specific restriction endonuclease sites. The sizes and localization of the restriction fragments after their electrophoretic separation are determined by Southern blotting [8]. MIRU-VNTR-typing uses *M*. *tuberculosis* genomic loci containing conservative tandem repeats as genetic markers (MIRU—Mycobacterial Interspersed Repetitive Units, VNTR—Variable Number Tandem Repeat). The number of these repeats is variable in different strains [9]. Besides these techniques, which are the most widespread, a number of additional approaches for *M*. *tuberculosis* genotyping are also available [6].

All these methods have their advantages and drawbacks [6]. Currently single nucleotide polymorphisms (SNPs) are considered the most promising genetic markers due to their low-level homoplasy and a high discriminating ability of genotyping techniques using SNPs. The main problem of these markers is the direct dependency of discriminating ability and the number of genes analyzed. Thus, the search for a set of genes with an optimal ratio of the number of loci and discriminating ability represents a significant task [10,11].

Type II toxin-antitoxin (TA) systems are widely spread among bacteria and archaea [12], including human commensal [13,14] and pathogenic bacteria [15,16]. Functions of type II TA systems are very diverse and actively studied [17,18]. It has been shown that this group of genes is involved in persistence regulation, biofilm formation, antibiotic tolerance, stress adaptation and virulence [19–23].

Type II TA systems represent a module of two genes, one after another, forming an operon. One of the genes encodes a stable toxin protein, the other one—a small labile antitoxin protein, which can bind to the toxin and inactivate it. Under stress conditions the antitoxin degradation occurs, leading to the toxin accumulation and cell growth inhibition [12].

Type II TA systems include a number of families, such as vapBC, relBE, mazEF, ccd, parDE, phd/doc, higBA, hipBA, which vary in their mode of action. Toxins belonging to families phd/doc, higBA, relBE, mazEF and vapBC are RNAses [24–28], toxins ParE and CcdB are DNA gyrases [29,30], HipA toxin phosphorylates elongation factor Tu, thus inhibiting peptide chain elongation [31].

The *M*. *tuberculosis* genome harbors a large number (from 70 to 90, according to various estimates) of type II TA systems, belonging to the following families: VapBC, MazEF, HigAB, RelBE and ParDE [32,33]. The functions of type II TA systems of *M*. *tuberculosis* are quite diverse [33], one of the most important being their involvement in virulence and transmissibility regulation [34, 35]. Correlation between SNPs in genes of TA systems type II of *M*. *tuberculosis* and belonging of the strains to a particular genotype, allows us to offer TA system as new markers for genotyping.

## Materials and Methods

### Bacterial strains and culture conditions

The bacterial strains used in this study are described in S1 Table. The *M*. *tuberculosis* strains were obtained from the collection of the Central TB Research Institute (CTRI), Moscow, Russia (http://www.cniitramn.ru/). *M*. *tuberculosis* cultures were cultured on an automatic growth detection system Bactec Mycobacterial Growth Indicator Tubes (MGIT) 960 (Becton Dickinson, Franklin Lakes, NJ, USA) according to the manufacturer's manual. Samples from Bactec MGIT 960 test tubes were plated on blood agar and incubated for 24 h at 37°C. If any growth was detected, the culture was considered contaminated and eliminated from the study.

### DNA manipulations

Genomic DNA was isolated from *M*. *tuberculosis* cultures on a robotized system EVO 150 (Tecan, Männedorf, Switzerland) with “M-Sorb-Tub-Avtomat” kit (Syntol, Moscow, Russia).

### Quantitative PCR (qPCR)

Detection of SNPs in TA genes was performed using a High Fidelity PCR Enzyme Mix (Fermentas) in a CFX96 thermocycler (Bio-Rad Laboratories Inc., Hercules, CA, USA). Probes and primers are described in S2 Table. Briefly, the PCR mixtures (final volume of 25 μl) contained 5 pmol each primer and probes, 1 ng cDNA, 5 mM dNTP mix, 2.5 μL of 10x PCR buffer with MgCl_2_ and RNase-free water to a final volume 25 μL. The thermal cycling conditions were as follows: 95°C for 5 min, then 35 cycles of denaturation at 94°C for 1 min, annealing at 64°C for 40 s and extension at 72°C for 40 s, and a final extension at 72°C for 10 min.

### Bioinformatic analysis

We analyzed 71 type II toxin-antitoxin systems belonging to five different families: VapBC, MazEF, HigAB, RelBE и ParDE (see S3 Table). We also included 3 «novel» TA systems that is not attributed to any family [32] (S3 Table). Gene sequences were obtained from GenBank (NCBI). The classification of putative toxin and antitoxins in families was performed according to Ramage et al. [33].

For our study 173 sequenced genomes were analyzed which were obtained from GenBank (NCBI), including 16 complete genomes, 84 genomes represented by contigs and 37 genomes represented by SRA archives (see S4 Table). The genomes with unknown genotype were classified according to the method of Homolka et al. [10].

All studied type II TA systems were checked for the presence of SNPs (TA systems from the *M*. *tuberculosis* H37Rv were taken as a reference). Polymorphisms (SNPs) and their locations were identified by BLAST software in conjunction with the Python scripts developed by us (see S5 Table). The program for genotyping strains was created based on the correlation of polymorphisms with genotypes.

### Phylogenetic analysis

To determine the position of genotypes and subtypes on the phylogenetic tree, we used nucleotide sequences of 71 type II TA systems (S3 Table) from 173 sequenced genomes of *M*. *tuberculosis* (S4 Table). Nucleotide sequences of 71 TA systems from each genome were concatenated in ‘supersequence’ (supergene), and the resulting 173 long supersequences were aligned with each other using Clustal W ver. 2.1. [36]. A phylogenic tree from the resulting alignment was constructed using Molecular Evolutionary Genetic Analysis (MEGA) software version 6 [37] by the neighbor-joining (NJ) method.

In addition, we constructed a phylogenetic tree based on the minimum set of genes, which we offer for genotyping *M*. *tuberculosis* strains. Thirteen genes were concatenated in supersequence (for each strain), and resulting 173 supersequences were aligned using ClustalW ver. 2.1. The phylogenetic tree was constructed using MEGA ver. 6 analogous to the large tree.

## Results and Discussion

### Genetic diversity of type II TA systems in *M*. *tuberculosis* genomes and the correlation of their genotypes with SNPs

A bioinformatics analysis of the genes of the type II TA systems from 173 sequenced genomes of *M*. *tuberculosis* showed that, out of 142 tested genes, 106 (74.6%) had polymorphisms (S5 Table). Missense SNPs were found in 42.2% cases, 29.0% were silent SNPs, 28.8% had insertions and deletions leading to a frameshift. Among missense SNPs originating in TA systems belonging to the VapBC family, most were localized in the functional part of the gene, the PIN-domain [38].

Along with the high degree of polymorphisms, the genes of the TA systems are partially conserved. The number of genes containing more than one SNP did not exceed 5% of examined genes. A homolog of the TA system VapBC31 (rv0749-rv0748) was found in all *M*. *tuberculosis* genomes and was annotated in the strain H37Rv as VapBC25 (rv0277c-rv0277A). The homology between those genes reached 89%; there was 93% homology for the toxin and 88% for the antitoxin. Presumably, the TA system VapBC25 (rv0277c-rv0277A) only partially functions or does not function at all because the SNP in 24 codons of the antitoxin becomes a stop codon. This system was excluded from further analysis.

**Fig 1:**
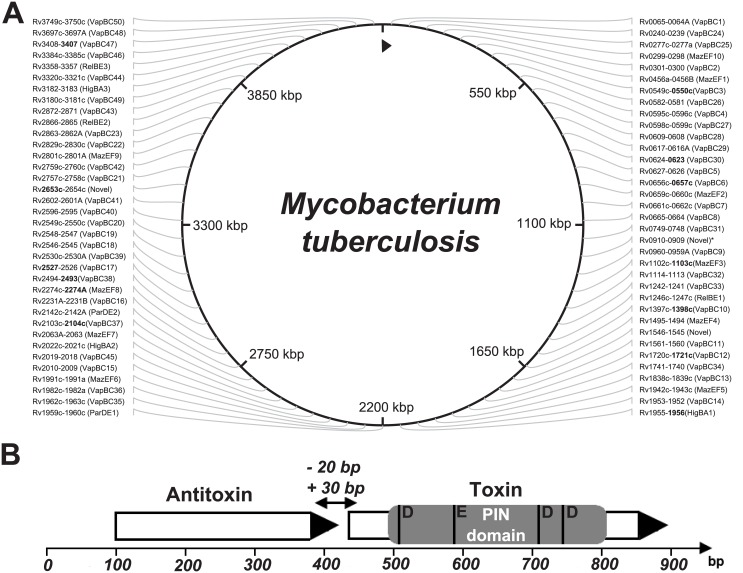
The type II TA systems of mycobacteria were investigated. Schematic diagram of the toxin-antitoxin system. (A) TA systems are annotated according to the GenBank database, excluding VapBC50 (rv3750c-rv3749c), VapBC49 (rv3180c-rv3181c), HigBA3 (rv3182-rv3183), HigBA2 (rv2022c-rv2021c), MazEF10 (rv0298-rv0299) and VapBC45 (rv2018-rv2019) systems; these systems are annotated according to Sala et al. [32]. The system RelBE3 (rv3358-rv3357, GenBank database, NCBI) is called the YefM/YoeB system by Sala. All of the TA systems depicted here are type II (systems marked with an asterisk are novel TA systems that are not classified to any family, but for which functional activity has been shown [32]). The 13 genes, our proposed set for genotyping, are highlighted in bold. (B) Type II TA systems are encoded by two genes, a toxin and an antitoxin, that form one operon with a promoter located upstream of the first antitoxin gene. PIN domain is the functional part of the toxin gene, the four conserved acidic residues marked at the picture: the three well-conserved acidic residues, at positions 4[D], 40[E] and 93[D], and with fourth acidic residue is less well conserved at position 112[D].

All 142 genes of the type II TA systems were found in the examined genomes except in two cases: the lack of the MazEF8 system (rv2274c-rv2274A) of the Haarlem genotype and the lack of the toxin gene (rv2760c) from that VapBC42 system, which were not found in strains NA-A0008 and NA-A0009 (EAI-Manila subtype, according to our classification, see S4 Table) (Fig 1).

For a number of type II TA systems, there were polymorphisms that correlated with a specific genotype. In summary, based on SNPs in TA systems, at first the *M*. *tuberculosis* genomes can be divided into two groups in accordance with the concept of global phylogeny of tuberculosis for the Euro-American lineage and non-Euro-American lineage, including the Indo-Oceanic lineage, East Asian lineage and East African-Indian lineage. The Indo-Oceanic lineage corresponds to the genotype EAI, East African-Indian lineage to genotype CAS, and East Asian lineage to the Beijing genotype [4]. In the Euro-American lineage, the following basic genotypes can be distinguished: Ural, X, Haarlem, LAM, S and T [5, 39]. Therefore, SNPs in the seven genes of the type II TA systems (*higA1*, *vapC6*, *vapC10*, *vapC38*, *mazF3*, *mazF8* и *vapC47*) allow for the identification of nine basic genotypes (Table 1).

**Table 1:** The division of Beijing genotype on subtypes using the polymorphisms in five genes belong to type II TA systems.

Subtype by Merker et al.^1^	Ancestral Asia 1–3 (CC6, BL7)		Typical/modern Beijing (CC1—CC5)	Europe-Russia W-148 (CC2)
Subtype	Beijing-like	Beijing- ancestral	Beijing-modern	B0/W-148
Gene	Genomes			
	CWCFVRF MDRTB 670, 94_M4241A	02_1987, 1034, XDR1221, NCGM2209, CTRI-4, R1207	210, WX1, WX3, T85, SP4, X122, XDR1219, SP6, SP11, SP5, SP9, SP16, SP18, SP2, MOS14, G-12-005, Beijing/NITR203, BT1, BT2, CCDC5079, CCDC5180, HKBS1, HN878, PanR0605, PanR0606, SP34, SP29, SP3, SP8, SP12, MOS12	SP1, SP10, SP21, SP22, SP7, W-148, SP13, MOS11
***mazF3***	194: C→T^2^	194: C→T	194: C→T	194: C→T
***higA1***	-	363: C→T	363: C→T	363: C→T
***vapC38***	-	-	143: T→C	143: T→C
***vapC37***	-	-	46: A→G	46: A→G
***vapC12***	-	-	-	95: A→G

^1^ The division into subtypes according to Merker [40].

^2^ The H37Rv strain was used as a reference.

Thus, using polymorphisms in seven genes of type II TA systems, we were able to index some of the nine basic genotypes by their subtype. Genotyping within the Beijing genotype has the most significant value to epidemiology.

According to modern concepts, the Beijing genotype was divided into the two most significant subtypes: modern and ancestral, which have differences in virulence and propensity for the formation of resistance [40–42]. The subtypes Beijing-modern and Beijing-ancestral, according to our classification, correspond to the following combination of SNPs: the *mazF3* and *higA1* genes, Beijing-ancestral subtype, and the *mazF3*, *higA1*, *vapC38* and *vapC37* genes, Beijing-modern subtype (Table 1).

With the addition of the *vapC12* gene, a cluster of B0/W-148 as part of the Beijing-modern subtype can be identified. This cluster has epidemiological significance and is endemic in Russia; however, it has become widespread [43,44].

According to our proposed classification, the strains 94_M4241A and CWCFVRF MDRTB 670 have SNPs in the *mazF3* (rv1102c) gene, and the lack of polymorphisms in the *higA1* (rv1956) gene, in connection with the SNPS in the *mazF3* (rv1102c) gene, allowed these strains to be allocated into a separate Beijing-like subtype. Strain 94_M4241A, according to the genotyping by Homolka et al. [10], belonged to the Beijing (Beijing-ancestral) subtype; however, according to recent studies, Homolka et al. took an intermediate position between the Beijing and EAI genotypes [45]. Strain CWCFVRF MDRTB 670, according to genotyping by housekeeping genes [10], belongs to the Beijing genotype.

Thus, the Beijing genotype, using the three additional genes, *vapC37*, *vapC12* and *vapC38*, can be divided into four subtypes: Beijing-modern, Beijing-ancestral, Beijing-like and endemic to the Russia cluster B0/W-148, which are associated with drug resistance and elevated virulence [3] (Table 2).

**Table 2:** The minimum set of genes for genotyping strains of *M*. *tuberculosis*, developed on the basis of SNP in genes of TA systems of VapBC, HigAB and MazEF families.

Genotype	Genes	SNP^1^	Subtype	Genes	SNP
**Beijing**	*mazF3 higA1 vapC47*	194:C→T 363:C→T 137:C→T	**Beijing-like**	*mazF3*	194:C→T
			**Beijing-ancestral**	*mazF3 higA1*	194:C→T 363:C→T
			**Beijing-modern**	*mazF3 higA1 vapC37 vapC38*	194:C→T 363:C→T 46:A→G 143:T→C
			**B0/W-148**	*mazF3 higA1 vapC37 vapC38 vapC12*	194:C→T 363:C→T 46:A→G 143:T→C 95:A→G
**Delhi/CAS**	*mazF3 vapC6 vapC47*	194:C→T 194:G→T 137: C→T	-	-	-
**EAI**	*mazF3 vapC10 vapC47*	194:C→T 308:A→G 137: C→T	**EAI-Manila**	*rv2653c* ^3^	111:C→T 272:A→T 294:C→G
**S**	*vapC6 vapC47*	280:G→A 137: C→T	-	-	-
**Haarlem**	*vapC38 vapC47 mazF8*	197:G→C 137:C→T No gene	-	-	-
**LAM**	*vapC47*	137:C→T	**LAM1**	*vapC30*	38:G→C
			**LAM2**	*vapC3*	222:G→A
			**LAM9**	*mazF8*	3:G→A
			**LAM4/F15/KZN**	*vapC38*	168:C→T
**T**	-	-^2^	**SMI-049**	*vapB17*	213:G→C
**Ural**	*vapC10 vapC47*	394:C→T 137:C→T	-	-	-
**X**	*vapC38 vapC47*	197:G→C 137:C→T	-	-	-

^1^ The H37Rv strain was used as a reference.

^2^ No polymorphisms relative to strain H37Rv because H37Rv relates to T genotype.

^3^ Novel family.

This classification is not final; with increasing amounts of type II TA systems, the discriminative power of the method increases. In particular, by adding two genes, *vapC1* and *higB1*, which are possible Beijing-ancestral subtype genes divided in three groups, and adding four genes, *mazF7*, *mazE3*, *vapC46* and *higB2*, which are possible Beijing-modern subtype genes divided in four groups, the cluster B0/W-148 is divided into three groups. In total, using polymorphisms in 11 genes, the Beijing genotype can be divided into 11 groups. Of particular interest is the division of the most important genes into subgroups, and in an epidemiological sense, the cluster of B0/W-148 is associated with an increased virulent phenotype because recent studies indicate that the composition of this cluster included strains with different phenotypes, including with reduced virulence [46].

The remaining genotypes were divided into the following subtypes: we distinguish the EAI-Manila subtype within the genotype EAI, using SNPs in the *rv2653* gene, a member of the family which has not yet been determined. The LAM genotype was divided into four subtypes corresponding with LAM1, LAM2, LAM9 and LAM4/F15/KZN by using polymorphisms in the following four genes: *vapC30*, *vapC3*, *vapC38* and *mazF8*. The T genotype can be distinguished into subtype SMI-049 using a polymorphism in the *vapB17* gene (Table 2). Earlier, the LAM4/F15/KZN and SMI-049 subtypes were identified by using RFLP-typing only [47,48].

Genes that are proposed to set genotyping are involved in the following known mechanisms: inhibition of the growth of *M*. *smegmatis* [33] (*mazF3*, *higA1*, *vapC10*, *vapC47*, *vapC37*, *rv2653c*, *vapC30*, *vapC3*), regulation of the growth rate (slowing) of *M*. *bovis* under stressful conditions [49] (*vapC37* and *vapC38*) and others (S3 Table).

### Phylogenetic relationship between different genotypes of the *M*. *tuberculosis* strains

**Fig 2:**
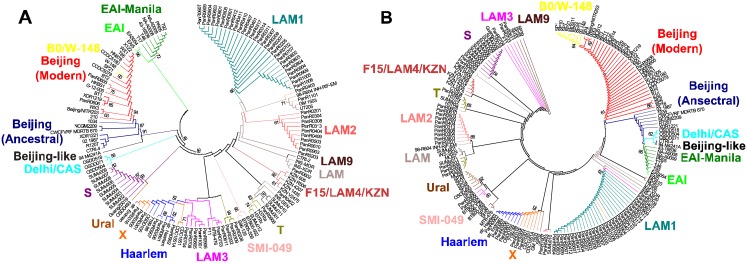
Phylogenetic relationship between different genotypes of the *M*. *tuberculosis*. (A) Phylogenetic tree constructed on the basis of polymorphisms (SNP) in all of the considered genes of type II TA systems. An unrooted phylogenetic tree for the 173 strains from this study was constructed based on the presence/absence of SNPs in the nucleotide sequences of 71 TA systems (S3 Table); (B) Phylogenetic tree constructed on the basis of SNP in a minimum set of genes of type II TA systems. An unrooted phylogenetic tree for 173 strains constructed based on SNPs in the nucleotide sequences of 13 genes (Table 2). In both of cases strains included in the one cluster belong to the same genotype (various genotypes highlighted by color). The trees was constructed by the neighbor-joining approach. The TA systems sequences were retrieved from different databases (see Materials and Methods). Sequences were multiply aligned by using ClustalW ver. 2.1 software. The trees was calculated using MEGA ver. 6. Bootstrap support > 60% is indicated for the trees.

To establish the phylogenetic relationship between different genotypes of *M*. *tuberculosis* strains, a set of nucleotide sequences of 71 type II TA systems (S3 Table) from 173 sequenced genomes of *M*. *tuberculosis* (S4 Table) were compared. These genes are highly conserved, but some of them have different SNPs conserved for the genotype, allows to divide genomes into clusters according to their genotypes. We aligned 71 TA systems (concatenated in the sequence) for each strain, and build the phylogenetic tree (Fig 2A). Additionally, we build a phylogenetic tree based on the minimum set of genes, which we offer for *M*. *tuberculosis* strains genotyping (Fig 2B).

Both phylogenetic trees indicated that the *M*. *tuberculosis* strains can be divided into nine basic genotypes (Table 2). Moreover, both trees showed clearly that the Beijing genotype may be subdivided into 4 large clusters, including Beijing-like, Beijing-modern, Beijing-ancestral and B0/W-148, and that the cluster of B0/W-148 located on the same branch with the cluster of Beijing-modern but this two clusters clearly separate; clusters of the Beijing and EAI genotypes located nearby, showing their evolutionary relationship. There are also clearly distinguishable clusters for the following genotypes and subtypes: S, Ural, Haarlem, X, LAM1, LAM2, SMI-049 and F15/LAM4/KZN. Most of the major internal nodes on the both trees had bootstrap support > 60%.

A phylogenetic tree built based on the 71 TA systems (Fig 2A) showed that the clustering of *M*. *tuberculosis* strains is almost identical to that obtained from the phylogenetic tree based on a minimum set of genes (Fig 2B). Thus, we can state that in the case of M. tuberculosis, genotyping based on the full set of genes generates groups similar to the one achieved by using a smaller number of genes.

On the PATRIC (Pathosystems Resource Integration Center, https://www.patricbrc.org/portal/portal/patric/Phylogeny?cType=taxon&cId=1763) website a phylogenetic tree for pathogenic actinobacteria, including pathogenic mycobacteria and *M*. *tuberculosis*, is available. The comparison of the trees based on SNPs in genes of TA systems and the tree from the PATRIC website showed high similarity—all three trees show the division of all the strains in two main lineages: Euro-American and non-Euro-American. The branch containing the Beijing genotype located near the one of the Delhi/CAS genotype, it can be more clearly observed on tree built on the minimal set of genes; the branch containing the EAI genotype (and EAI-Manila subtype) can be clearly identified on all three trees. Inside the Euro-American lineage clusters of the following genotypes and subtypes can be determined quite distinctly: S, LAM1, Haarlem, X и SMI-049. Moreover, Haarlem and X genotypes are nested on one the branch in all of the three trees. Strains of the Ural genotype are absent on the tree from the PATRIC website.

Some common features can be also observed in the comparison of our phylogenetic trees with the trees published by Homolka et al., [10]: the EAI genotype is localized on a branch separated from the branch harboring Beijing and Delhi/CAS genotypes; X, LAM1, S, Ural and Haarlem are clearly divided and belong to the Euro-American lineage.

The phylogenetic analysis confirms our findings, that we can genotype mycobacterial strains using a set of genes of TA systems with significant SNPs, and that our results can not only divide the strains at the nine basic genotypes, but also allow to identify subtypes within them.

### Polymorphisms of type II TA system genes: a new set for genotyping

The correlation between a strain belonging to a particular genotype and the presence of SNPs in different genes of type II TA systems allows us to consider the TA systems as a new functional bio-marker for genotyping *M*. *tuberculosis*. Currently, there are several different genotyping methods that differ by a variety of markers, that is, DR-repeats, VNTR (variable number tandem repeats) and an IS*6110*-element [6]. Recently the interest emerged to SNPs, in particular, in housekeeping genes, as markers for genotyping. The advantage of SNPs is the low level of homoplasy and an unambiguous interpretation; the disadvantage is a reduced discriminating ability and a decreased number of genes that can be used for genotyping.

**Fig 3:**
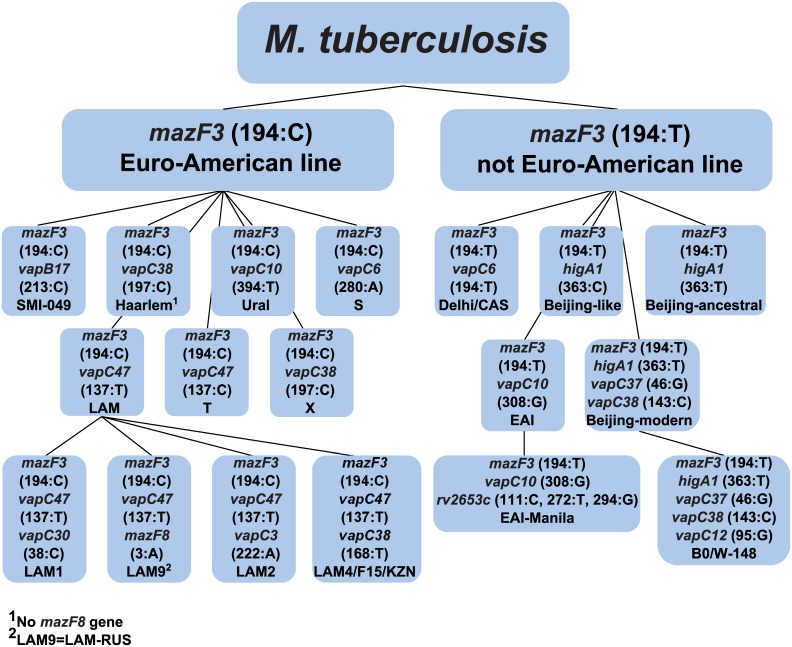
Scheme of typing of *M*. *tuberculosis* strains using 13 genes of type II TA systems. The algorithm for determining the genotype is presented. The scheme shows that, after the first iteration to determine the genotype, the number of genes for the analysis is decreased twofold. Each gene in the brackets is given its position that is replaced, and the appropriate nucleotide is indicated. All replacements are calculated relative to the reference strain H37Rv.

When using SNPs in type II TA systems with a minimum number of genes (13 genes), all basic groups of *M*. *tuberculosis* can be represented, including those with clinical significance, such as the LAM4/F15/KZN and B0/W-148 subtypes; the Beijing genotype can be divided into four subtypes. Fig 3 shows a scheme for genotyping using 13 genes of the type II TA systems. The following algorithm is proposed, which reduces the number of genes that need to be used: 1) a SNP in the *mazF3* gene classified *M*. *tuberculosis* into two lineages, including the Euro-American and not Euro-American lineages; 2) further, if the *mazF3* gene has a thymine (T) at the position 194, the following seven genes should be used: *vapC6*, *higA1*, *vapC10*, *vapC37*, *vapC38*, *rv2653c* and *vapC12*; 3) and if the *mazF3* gene has a cytosine (C) at the position 194, the following eight genes should be used: *vapC6*, *vapC10*, *vapC30*, *vapB17*, *vapC38*, *vapC3*, *vapC47* and *mazF8*. Therefore, in both cases, the number of genes is reduced to nine, without the loss of resolution.

After the completion of the work with the GenBank database we randomly took 10 additional genomes. These genomes were genotyped in two ways: using SNPs in the housekeeping genes, and using the SNPs in the genes belonging to the type II TA systems (Table 3).

**Table 3:** Determination of genotypes of tested strains in two ways.

Strain	Method is based on the	
	TA genes	Housekeeping genes
**2484AR**	LAM	not defined^1^
**49–02**	Beijing- modern	Beijing
**96075**	Beijing-modern	Beijing
**96121**	EAI-Manila	EAI-Manila
**K**	Beijing	Beijing
**KIT87190**	Beijing	Beijing
**Kurono**	LAM	not defined^1^
**MD17898**	Beijing-modern	Beijing
**ZMC13-264**	Beijing	Beijing
**ZMC13-88**	Beijing	Beijing

^1^ These two genomes belong to the Euro-American lineage according to SNPs in the housekeeping genes.

As shown in Table 3, the genotypes determined by both methods coincided, but the method of SNPs in the TA system genes allows to determine the subtype along with the genotype. All the above confirms that the genotyping method proposed by us can be applied to any genome to accurately indicate the genotype/subtype of the microorganism.

### Comparative genotyping of isolate collection

Using the proposed set of markers, we genotyped a collection of 62 clinical isolates of *M*. *tuberculosis*, including three groups that differ in drug resistance and clinical manifestations [50]. For each isolate, the sequence of the 13 genes was determined. As a reference, the method [10] was used. We identified 43 isolates belonging to the Beijing-modern subtype (including 16 isolates of the B0/W-148 subtype), 1 isolate belonging to the X genotype, 2 isolates with the Ural genotype and 16 isolates belonging to the LAM genotype (including one belonging to the LAM9 subtype, Table 4).

**Table 4:** Comparative genotyping of the Russian collection of isolates by two methods.

Genotype	Genotyping using housekeeping genes	Genotyping using genes of toxin-antitoxin systems	
		Genotype	Subtype
**Beijing**	43	43	16 *(B0/W-148)* 27 *(Beijing-modern)*
**Ural**	2	2	-
**LAM**	7	16	1 *(LAM9)* 15 *(LAM)*
**X**	0	1	-
**Unknown strain of Euro-American lineage**	10	0	-

### Detection of polymorphisms using qPCR

We carried out genotyping of 62 clinical isolate of *M*. *tuberculosis* using the qPCR and the proposed set of genes. As a reference, the H37Rv strain (belongs to T genotype of the Euro-American lineage) was used. The absence of an SNP (the position is same as the reference strain) was detected via FAM (blue) channel, while the presence of SNPs was detected via HEX (green) channel.

At first, according to Fig 3, all isolates were divided into two groups: Euro-American and non-Euro-American. Isolates belonging to the Euro-American lineage had cytosine in the *mazF3* gene in the position 194 (no substitution compared to the reference) whereas the isolates of the non-Euro-American lineage had thymine (substitution compared to the reference). Thus, 43 isolates were identified as the non-Euro-American lineage, and 19 as the Euro-American lineage.

All 43 isolates belonging to the non-Euro-American lineage had a polymorphism in the *higA1* gene (C_363_→T_363_), and therefore belonged to the Beijing genotype. With further genotyping using SNP in *vapC37*, *vapC38* and *vapC10* genes these isolates were divided into two groups: 27 belonged to Beijing-modern genotype and 16 were attributed to B0/W-148 genotype.

Among the isolates of the Euro-American lineage one belonged to X genotype, 2 isolates to the Ural genotype, and 15 to the LAM genotype (including one of the LAM9). All the results obtained by PCR were consistent with the data presented in Table 4. Therefore, polymorphisms as the markers for a particular genotype can be identified not only by sequencing but by a less laborious qPCR technique.

**Fig 4:**
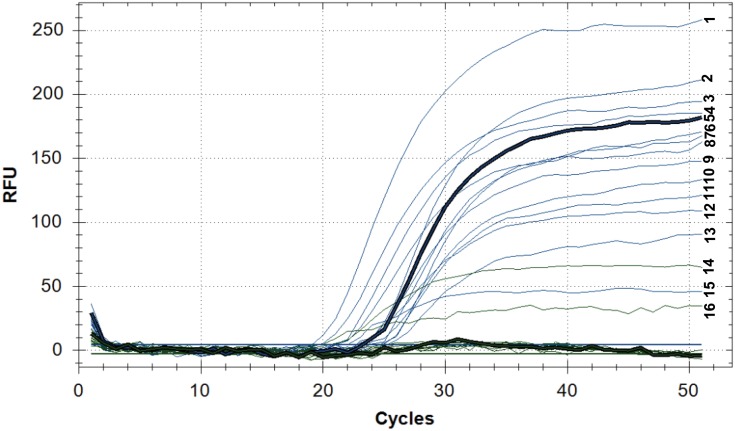
Detection of the Ural genotype by qPCR. Fluorescence in the FAM channel (blue): (1)13_2978, (2) 13–3114, (3) 13–3086, (4) 13_3158, (5) 13_4178, (6) 13_3539, (7) 13_2566, (8) 13_3632, (9) 13_3599, (10) 13_3896, (11) 13_3582, (12) 13_4189, (13) 13_3535, (15) 13_3147; Fluorescence in the HEX channel (green): (14) 13_3147, (16) 13_2978. Fluorescence of the channel FAM (blue) indicates the accumulation of the PCR product containing cytosine (C); the fluorescence of the channel HEX (green) indicates the accumulation of the PCR product containing thymine (T, the variable nucleotide) and indicates the SNP in the *vapC10* gene (C394→T394) characteristic of the Ural genotype. Line 14 (13_3147) and 16 (13_2978) belong to the Ural genotype. For isolate 13_2978 fluorescence is detected on the two channels (FAM and HEX), this can indicate the presence of impurities (coinfection). qPCR fluorescence in RFU (relative fluorescence units) vs. PCR cycles. Intensity of fluorescence depending on the number of qPCR cycles for strains belonging to the Euro-American lineage.

Fig 4 shows an example of the Ural genotype detection by qPCR. Two strains 13–3147 and 13_2978 belonging to the Ural genotype were identified in the Euro-American lineage. This strain carried a substitution (C394→T394) in the *vapC10* gene, typical for the Ural genotype.

## Concluding Remarks

We propose an alternative approach for genotyping of *M*. *tuberculosis* strains based on SNPs in type II TA systems. Type II TA systems play an important role in the pathogen’s adaptation to adverse conditions and SNPs in these genes may potentially alter the activity of the encoded proteins. Two phylogenetic trees were constructed. One is based on SNPs identified in the 71 type II TA systems, and another one on the basis of SNPs in 13 genes included in the set for genotyping. Both phylogenetic trees break down into identical clusters, confirming a possibility of using a minimal set of genes to divide the M. tuberculosis strains into genotypes and subtypes. Thus, a set of 13 genes of the type II TA system was developed, and using this set of genes, 62 clinical isolates were genotyped. A program for genotyping was created based on SNPs in genes of TA systems. The possibility of using our set of genes for genotyping using PCR has been also demonstrated. Investigations of the functional roles of the detected polymorphisms in type II TA systems are of great interest because different strains of *M*. *tuberculosis* (and, respectively, different genotypes) may adapt to the host conditions with different efficiency. SNPs of TA systems and other groups of genes associated with virulence and pathogenicity require further systematization and research.

## Supporting Information

S1 Table DNA Samples used in this study. (PDF)

S2 Table Primers for cloning of toxin genes used in this study. (PDF)

S3 Table Type II toxin-antitoxin systems of *M*. *tuberculosis* discussed in this article. (PDF)

S4 Table Genomes of *M*. *tuberculosis* from the international database GenBank (NCBI) collected and investigated in this article. (PDF)

S5 Table Type II toxin-antitoxin systems of *M*. *tuberculosis* discussed in this article (112K, xls). (XLS)
