# Supplementary material for: Mycobacterium tuberculosis Type II Toxin-Antitoxin Systems: Genetic Polymorphisms and Functional Properties and the Possibility of Their Use for Genotyping
Source: PLoS One. 2015 Dec 14;10(12):e0143682. doi: 10.1371/journal.pone.0143682 (PMC4680722; doi:10.1371/journal.pone.0143682)
Supplement: S1 Table — (PDF) [file pone.0143682.s001.pdf]

**S1 Table. DNA Samples used in this study.**

| Name | Description | Source |
|---|---|---|
| **Strains (*M. tuberculosis*)** | | |
| H37Rv | Purified genome DNA of virulent laboratory strain H37Rv | Central Research Institute of Tuberculosis, Moscow, Russia (http://www.cniitramn.ru) |
| Mos10 | Purified genome DNA of clinical isolate Mos10 | Scientific Research Institute of Physical-Chemical Medicine, Moscow, Russia (http://niifhm.ru/home/) |
| 13-4189, 13-4178, 13-3935, 13-3896, 13-3632, 13-3617, 13-3594, 13-3582, 13-3539, 13-3158, 13-3055, 13-2566, 13-2292, 13-1870, 13-2978, 13-3086, 13-3114, 13-3147, 13-3208, 13-4446, 13-4220, 13-4050, 13-4009, 13-3629, 13-3538, 13-3537, 13-3387, 13-3292, 13-3085, 13-2975, 13-2041, 13-2078, 13-2105, 13-2243, 13-2385, 13-2392, 13-2432, 13-2674, 13-2776, 13-3134, 13-3435, 13-3636, 13-3805, 13-4064, 13-4152, 13-4177, 13-4614, 13-1871, 13-1936, 13-1965, 13-2253, 13-2376, 13-2459, 13-2836, 13-2864, 13-3318, 13-3373, 13-3390, 13-3742, 13-3917, 13-3918, 13-4781 | Purified genome DNA of 62 clinical isolate | Central Research Institute of Tuberculosis, Moscow, Russia (http://www.cniitramn.ru) |
