# Supplementary material for: Mycobacterium tuberculosis Type II Toxin-Antitoxin Systems: Genetic Polymorphisms and Functional Properties and the Possibility of Their Use for Genotyping
Source: PLoS One. 2015 Dec 14;10(12):e0143682. doi: 10.1371/journal.pone.0143682 (PMC4680722; doi:10.1371/journal.pone.0143682)
Supplement: S2 Table — (PDF) [file pone.0143682.s002.pdf]

S2 Table. Primers for cloning of toxin genes used in this study.

Locus tag	Gene	Primer	Sequence (5'- 3')	Product size (bp)	Reference
**Rv2103c**	***vapC37***	Rv2103cN	GGACGAAGAGCTTGTGCGCCGTCA	181	This study
		Rv2103cC	CAACAACGGCACCCAGGCGAACCC		This study
**Rv1956**	***higA1***	Rv1956-N	CTGCGGCTGGTGCTCGAAGTTCCCA	213	This study
		Rv1956-C	CGCGTTGCAGGTCAAGGTCGTC		This study
**Rv2494**	***vapC38***	Rv2494-N1	GATCCGCGAGTGGTTTACCGCCAA	194	This study
		Rv2494-C1	ACGAGCGATACGTCGTCAGCCAG		This study
**Rv1397c**	***vapC10***	Rv1397cdir	CGTGTGGCATTTCCCCATGTTCGAG	192	This study
		Rv1397crev	AGGAGTGCGGTCTTATTGCAAC		This study
		Rv1397cN	AACCGGATTGGACTTGGCGACTA	244	This study
		Rv1397cC	CTTTGTTTATTAATGTCGCGCGGCCC		This study
**Rv2274c**	***mazF8***	Rv2274cN	CGGTGTTGAGGTCAGTGATCAGGGT	138	This study
		Rv2274cC	GTGAGTGTACCCGCCGCCACAG		This study
**Rv3408**	***vapC47***	Rv3408N	GACACCTCGGCCCTGACTAAGCTG	225	This study
		Rv3408C	GATCACCGGTTCGGTGAGCGGGA		This study
**Rv1102c**	***mazF3***	mazF3N	TGACAAGGCTAGACCCGTCCTGAT	218	This study
		mazFC	CGAGCAGGTAGCCGATTTGACGA		This study
**Rv1720c**	***vapC12***	Rv1720cdir	GCGCAGCCGAGCTTGTTGACGAGG	246	This study
		Rv1720crev	ACGGGCAGGCTAAGGAAGTTCACA		This study
**Rv0656c**	***vapC6***	Rv0656c-N	GTGCCCTGTCGGTTGTCGATCTGCT	192	This study
		Rv0656c- C	ATCAGCCATGCGTGTCTCCTTGTCC		This study
		Rv0656cNb	GAGTTCGACCAGATGAGCCGGATG	194	This study
		Rv0656cCb	GCATCGTCGTGGAGGACTACTAGG		This study
**Rv2526**	***vapB17***	Rv2526-N1	TCGTCGACCATCTCGCGCACG	194	This study
		Rv2526-C1	AATACAGCCATTCAAGTTCGCCGAT		This study
**Rv0624**	***vapC30***	06241N	AGACGTTGACCGAGGCAGTGGTTA	375	This study
		06241C	CGATGAAGCCACAGATCCAGCTCA		This study
**Rv0549c**	***vapC3***	05492N	GACGAAAGACCGTGTCGAGTCACC	382	This study
		05492C	GAGTTGGAGAACTCCGCAACCGAT		This study
**Rv2653c**	***rv2653c***	NovelN	ACCGTCGATCACATCATTTTCCAGG	255	This study

		NovelC	TTCGGCTGTGTCGTCGTCGATTTC		This study
**Probes for Real-Time PCR**					
**Rv1397c**	***vapC10***	Rv1397U	(HEX)- 5’-GCACCGACCGTGGGCATGACA-3’-(BHQ2)	-	This study
		Rv1397nU	(FAM)- 5’-CACCGACCGCGGGCATGACA-3’-(RTQ1)		This study
**Rv1720c**	***vapC12***	Rv1720cW	(FAM)- 5’-GAAACCGTGCACGCCCCTGCA-3’-(RTQ1)	-	This study
		Rv1720cnW	(HEX)- 5’-GAAACCGTGCGCGCCCCTGCA-3’- (BHQ2)	-	This study
**Rv1956**	***higA1***	Rv1956nB	(FAM)- 5’-CACGGGACCACCAGGTCCGGG 3’- (RTQ1)	-	This study
		Rv1956B	(HEX)- 5’-CACGGGACCATCAGGTCCGGG 3’- (BHQ2)	-	This study
**Rv2494**	***vapC38***	Rv2494nB	(FAM)- 5’-TTCGTGCGGGTGTCGACGAA-3’- (RTQ1)	-	This study
		Rv2494B	(HEX)- 5’-TTCGTGCGGGCGTCGACGAA-3’- (BHQ2)	-	This study
**Rv2103c**	***vapC37***	Rv2103nB	(FAM)- 5’-CGTGAACACCACAAGTGAGCA 3’-(RTQ1)	-	This study
		Rv2103B	(HEX)- 5’-CGTGAACACCGCAAGTGAGCA 3’-(BHQ2)	-	This study
**Rv1102c**	***mazF3***	mazF3EAL	(FAM)- 5’-TGCGACAACACCCAAACGA-3’-(RTQ1)	-	This study
		mazF3nEAL	(HEX)- 5’-TGCGACAACATCCAAACGA 3’-(BHQ2)	-	This study
