# Supplementary material for: Mycobacterium tuberculosis Type II Toxin-Antitoxin Systems: Genetic Polymorphisms and Functional Properties and the Possibility of Their Use for Genotyping
Source: PLoS One. 2015 Dec 14;10(12):e0143682. doi: 10.1371/journal.pone.0143682 (PMC4680722; doi:10.1371/journal.pone.0143682)
Supplement: S3 Table — (PDF) [file pone.0143682.s003.pdf]

S3 Table. Type II toxin-antitoxin systems of *M. tuberculosis* discussed in this article.

№	Family	Toxin-antitoxin system	Toxin	Antitoxin	Functions
			(locus_tag)		
**1**	ND[1]	Novel	rv1546	rv1545	Not inhibit the growth of *M. smegmatis*[6]
**2**			rv2653c	rv2654c	Inhibit the growth of *M. smegmatis*[6]
**3**			rv0910	rv0909	Inhibit the growth of *M. smegmatis*, but the mechanism is unknown[6]
**4**	MazEF	mazEF1	rv0456A	rv0456B	Not inhibit the growth of *M. smegmatis*[6]
**5**		mazEF2	rv0659c	rv0660c	
**6**		mazEF3	rv1102c	rv1103c	Inhibit the growth of *M. smegmatis*[6]
**7**		mazEF4	rv1495	rv1494	Not inhibit the growth of *M. smegmatis*[6]
**8**		mazEF5	rv1942c	rv1943c	Inhibit the growth of *M. smegmatis*[6]
**9**		mazEF6	rv1991c	rv1991A	
**10**		mazEF7	rv2063A	rv2063	
**11**		mazEF8	rv2274c	rv2274A	Not tested
**12**		mazEF9	rv2801c	rv2801A	Inhibit the growth of *M. smegmatis*[6]
**13**		mazEF10[2]	rv0299	rv0298	
**14**	RelBE	relBE1	rv1246c	rv1247c	
**15**		relFG (relBE2)	rv2866	rv2865	
**16**		relJK (relBE3)[3]	rv3358	rv3357	Inhibit the growth of *M. smegmatis*[4]
**17**	ParDE	parDE1	rv1959c	rv1960c	Not inhibit the growth of *M. smegmatis*[6]
**18**		parDE2	rv2142c	rv2142A	Not tested for *M. smegmatis*, but inhibit the growth of *E. coli*[4]
**19**	HigAB	higAB1	rv1955	rv1956	Inhibit the growth *M. smegmatis*, expression in hypoxic conditions[6]
**20**		higAB2[2]	rv2022c	rv2021c	Not tested
**21**		higAB3[2]	rv3182	rv3183	Not inhibit the growth of *M. smegmatis*[4]
**22**	VapBC	vapBC1[2]	rv0065	rv0064A	

**23**		vapBC2	rv0301	rv0300	Inhibit the growth of *M. smegmatis*, by disrupting translation due to destruction of RNA[6]
**24**		vapBC3	rv0549c	rv0550c	Inhibit the growth of *M. smegmatis;* induction in macrophages; induced by hypoxia[6], SDS stress[7], during adaptation to nutrient starvation[12]
**25**		vapBC4	rv0595c	rv0596c	Not inhibit the growth of *M. smegmatis*[6]; induced during adaptation to nutrient starvation[12], macrophage infection[11] and by SDS stress[7]
**26**		vapBC5	rv0627	rv0626	Not inhibit the growth of *M. smegmatis*[6] or *E. coli*[13], but inhibit the growth of *M. tuberculosis*; toxin has RNase activity[14]
**27**		vapBC6	rv0656c	rv0657c,	Not inhibit the growth of *M. smegmatis*[6]
**28**		vapBC7	rv0661c	rv0662c	
**29**		vapBC8	rv0665	rv0664	
**30**		vapBC9	rv0960	rv0959A	
**31**		vapBC10	rv1397c	rv1398c	Inhibit the growth of *M. smegmatis*[6]
**32**		vapBC11	rv1561	rv1560	Inhibit the growth of *M. smegmatis*, by disrupting translation due to destruction of RNA; induction in macrophages[6]
**33**		vapBC12	rv1720c	rv1721c	Not inhibit the growth of *M. smegmatis*[6]
**34**		vapBC13	rv1838c	rv1839c	Inhibit the growth of *M. smegmatis*[6]
**35**		vapBC14	rv1953	rv1952	Not inhibit the growth of *M. smegmatis*[6]
**36**		vapBC15	rv2010	rv2009	Inhibit the growth *M. smegmatis*, expression in hypoxic conditions[6], but repressed during nutrient starvation[8],adaptation to hypoxia[9].
**37**		vapBC16	rv2231A	rv2231B	Not inhibit the growth of *M. smegmatis*[6]
**38**		vapBC17	rv2527	rv2526	
**39**		vapBC18	rv2546	rv2545	
**40**		vapBC19	rv2548	rv2547	Inhibit the growth of *M. smegmatis*[6]; induced during hypoxia[10] and macrophage infections[11], but repressed

					in sputum[15]
**41**		vapBC20	rv2549c	rv2550c	Not inhibit the growth of *M. smegmatis*[6]; induced during macrophage infection[11]
**42**		vapBC21	rv2757c	rv2758c	Inhibit the growth of *M. smegmatis*[6]
**43**		vapBC22	rv2829c	rv2830c	Inhibit the growth of *M. smegmatis*, by disrupting translation due to destruction of RNA[6]; induced during macrophage infection, hypoxia[6,9] and nutrient starvation[12]
**44**		vapBC23	rv2863	rv2862A	Not inhibit the growth of *M. smegmatis*[6]
**45**		vapBC24	rv0240	rv0239	
**46**		vapBC25	rv0277c	rv0277A	Inhibit the growth of *M. smegmatis*[6]
**47**		vapBC26	rv0582	rv0581	
**48**		vapBC27	rv0598c	rv0599c	Not inhibit the growth of *M. smegmatis*[6]
**49**		vapBC28	rv0609	rv0608	Inhibit the growth of *M. smegmatis*[6]
**50**		vapBC29	rv0617	rv0616A	Not inhibit the growth of *M. smegmatis*[6]
**51**		vapBC30	rv0624	rv0623	Inhibit the growth of *M. smegmatis*[6]
**52**		vapBC31	rv0749	rv0748	
**53**		vapBC32	rv1114	rv1113	
**54**		vapBC33	rv1242	rv1241	
**55**		vapBC34	rv1741	rv1740	Not inhibit the growth of *M. smegmatis*[6]
**56**		vapBC35	rv1962c	rv1962A	Inhibit the growth of *M. smegmatis*[6]
**57**		vapBC36	rv1982c	rv1982A	Not inhibit the growth of *M. smegmatis*[6]
**58**		vapBC37	rv2103c	rv2104c	Inhibit the growth of *M. smegmatis*[6]; involved in the regulation of the growth rate (slowing) *M. bovis* under stressful conditions[5]
**59**		vapBC38	rv2494	rv2493	Not inhibit the growth of *M. smegmatis*[6], but involved in the regulation of the growth rate (slowing) *M. bovis* under stressful conditions[5]
**60**		vapBC39	rv2530c	rv2530A	Inhibit the growth of *M. smegmatis*[6]

**61**		vapBC40	rv2596	rv2595	Not inhibit the growth of *M. smegmatis*[6]
**62**		vapBC41	rv2602	rv2601A	Inhibit the growth of *M. smegmatis*[6]
**63**		vapBC42	rv2759c	rv2760c	Not inhibit the growth of *M. smegmatis*[6]
**64**		vapBC43	rv2872	rv2871	Inhibit the growth of *M. smegmatis*[6]
**65**		vapBC44	rv3320c	rv3321c	Not inhibit the growth of *M. smegmatis*[6]; repressed during hypoxia[9] and nutrient starvation[8]
**66**		vapBC45[2]	rv2019	rv2018	Not tested
**67**		vapBC46	rv3384c	rv3385c	Inhibit the growth of *M. smegmatis*[6]
**68**		vapBC47	rv3408	rv3407	Inhibit the growth of *M. smegmatis*, by disrupting translation due to destruction of RNA[6]
**69**		vapBC48	rv3697c	rv3697A	Not tested
**70**		vapBC49[2]	rv3180c	rv3181c	
**71**		vapBC50[2]	rv3749c	rv3750c	

[1]ND = not determined
[2]These systems are described and annotated by Sala et al. in 2014 [1].
[3]Annotation of this system at the GenBank (sequences database, NCBI) looks like a relBE3, but Sala P. [1] proposed to call it YefM/YoeB.
[4] According to Sala P. et al [1].
[5] According Beste DJ et al. [2].
[6] According Ramage HR et al. [3].
[7] According Manganelli R et al. [4].
[8] According Betts JC et al. [5].
[9] According Sherman DR et al. [6].
[10] According Muttucumaru DG et al. [7].
[11] According Stewart GR et al. [8].
[12] According Hampshire T et al. [9].
[13] According Gupta A et al. [10].
[14] According Miallau L et al. [11].
[15] According Garton NJ et al. [12].

References:

1. Sala A, Bordes P, Genevaux P. Multiple toxin-antitoxin systems in *Mycobacterium tuberculosis*. Toxins (Basel). 2014 Mar; 6(3):1002-20. doi: 10.3390/toxins6031002.
2. Beste DJ, Espasa M, Bonde B, Kierzek AM, Stewart GR, McFadden J. The genetic requirements for fast and slow growth in mycobacteria. PLoS One. 2009 Apr; 4(4):e5349. doi: 10.1371/journal.pone.0005349.
3. Ramage HR, Connolly LE, Cox JS. Comprehensive functional analysis of *Mycobacterium tuberculosis* toxin-antitoxin systems: implications for pathogenesis, stress responses, and evolution. PLoS Genet. 2009 Dec; 5(12):e1000767. doi: 10.1371/journal.pgen.1000767.
4. Manganelli R, Voskuil MI, Schoolnik GK, Smith I. *Mycobacterium tuberculosis* ECF sigma factor sigmaE: role in global gene expression and survival in macrophages. Mol Microbiol. 2001 Jul; 41(2):423-37. doi: 10.1046/j.1365-2958.2001.02525.x.
5. Betts JC, Lukey PT, Robb LC, McAdam RA, Duncan K. Evaluation of a nutrient starvation model of *Mycobacterium tuberculosis* persistence by gene and protein expression profiling. Mol Microbiol. 2002 Feb; 43(3):717-31. doi: 10.1046/j.1365-2958.2002.02779.x.
6. Sherman DR, Voskuil M, Schnappinger D, Liao R, Harrell MI, Schoolnik GK. Regulation of the *Mycobacterium tuberculosis* hypoxic response gene encoding alpha-crystallin. Proc Natl Acad Sci USA. 2001 Jun; 98: 7534-7539. doi: 10.1073/pnas.121172498.
7. Muttucumaru DG, Roberts G, Hinds J, Stabler RA, Parish T. Gene expression profile of *Mycobacterium tuberculosis* in a non-replicating state. Tuberculosis (Edinb). 2004; 84(3-4): 239-246. doi: http://dx.doi.org/10.1016/j.tube.2003.12.006.
8. Stewart GR, Patel J, Robertson BD, Rae A, Young DB. Mycobacterial mutants with defective control of phagosomal acidification. PLoS Pathog. 2005 Nov; 1(3): 269-278. doi: 10.1371/journal.ppat.0010033.
9. Hampshire T, Soneji S, Bacon J, James BW, Hinds J, Laing K, et al. Stationary phase gene expression of *Mycobacterium tuberculosis* following a progressive nutrient depletion: a model for persistent organisms? Tuberculosis (Edinb). 2004; 84(3-4):228-38. doi: 10.1016/j.tube.2003.12.010.
10. Gupta A. Killing activity and rescue function of genome-wide toxin-antitoxin loci of Mycobacterium *tuberculosis*. FEMS Microbiol Lett. 2009 Jan; 290(1):45-53. doi: 10.1111/j.1574-6968.2008.01400.x.
11. Miallau L, Faller M, Chiang J, Arbing M, Guo F, Cascio D, et al. Structure and proposed activity of a member of the VapBC family of toxin-antitoxin systems. VapBC-5 from *Mycobacterium tuberculosis*. J Biol Chem. 2009 Jan; 284(1):276-83. doi: 10.1074 /jbc.M805061200.
12. Garton NJ, Waddell SJ, Sherratt AL, Lee SM, Smith RJ, Senner C, et al. Cytological and transcript analyses reveal fat and lazy persister-like bacilli in tuberculous sputum. PLoS Med. 2008 Apr ; 5(4):e75. doi: 10.1371/journal.pmed.0050075.
