# Supplementary material for: Mycobacterium tuberculosis Type II Toxin-Antitoxin Systems: Genetic Polymorphisms and Functional Properties and the Possibility of Their Use for Genotyping
Source: PLoS One. 2015 Dec 14;10(12):e0143682. doi: 10.1371/journal.pone.0143682 (PMC4680722; doi:10.1371/journal.pone.0143682)
Supplement: S4 Table — (PDF) [file pone.0143682.s004.pdf]

S4 Table. Genomes of *M. tuberculosis* from the international database GenBank (NCBI) collected and investigated in this article.

№	Genotype	Strains	Origin	Accession number	Assembly level
1	Beijing (Modern)	HN878	USA	ADNF01000000	Chromosome
2		T85	USA (Chinese migrants)	ABOW00000000	Scaffold
3		210	USA	ADAB00000000	Contig
4		CCDC5079	China	CP001641	Complete Genome
5		X122	Western Cape of South Africa	ADNG00000000	Chromosome
6		CCDC5180	China	CP001642	Complete Genome
7	Beijing (Ancestral)	R1207	Western Cape of South Africa	ADNH00000000	Chromosome
8		CTRI-4	Tomsk, Russia	AIIE01000000	Contig
9		02_1987	USA (ethnic, Korean)	ABLM00000000	Scaffold
10		94_M4241A	USA (ethnic, Chinese)	ABLL00000000	Scaffold
11	Beijing	SP2	Russia	SRX216884	Reads
12		SP3		SRX216885	
13		SP4		SRX216886	
14		SP5		SRX216887	
15		SP6		SRX216888	
16		SP8		SRX216890	
17		SP9		SRX216891	
18		SP11		SRX216893	
19		SP12		SRX216894	
20		SP16		SRX216896	
21		SP18		SRX216898	
22		SP29		SRX216904	
23		MOS10		SRX216917	
24		MOS12		SRX216919	
25		MOS14		SRX216920	
26		XDR1221	China	AJGN00000000	Contig
27		XDR1219		AJGO00000000	Contig
28		WX3		AJGL00000000	Contig
29		WX1		AJGM00000000	Contig
30		Beijing/ NITR203	India	CP005082	Complete

					Genome
**31**		PanR0606	Panama	ATRX00000000	Chromosome
**32**		PanR0605		ATRW00000000	Chromosome
**33**		NCGM2209	Japan	BADQ00000000	Scaffold
**34**		CWCFVRF MDRTB 670	India	JDVY00000000	Contig
**35**		HKBS1	N/A (Beijing/ W Lineage)	CP002871	Complete Genome
**36**		G-12-005	Georgia	JHUF00000000	Contig
**37**		BT2	N/A (Beijing/W)	CP002882	Complete Genome
**38**		BT1	N/A (Beijing/W)	CP002883	Complete Genome
**39**		1034	China	APHO00000000	Contig
**40**	B0/W-148	SP1	Russia	SRX216883	Reads
**41**		SP7		SRX216889	
**42**		SP10		SRX216892	
**43**		SP21		SRX216899	
**44**		SP13		SRX216895	
**45**		SP22		SRX216900	
**46**		MOS11		SRX216918	
**47**		W-148		ACSX00000000	Scaffold
**48**	EAI	EAS054	USA (Born in India)	ABOV00000000	Scaffold
**49**		EAI5	India	CP006578	Complete Genome
**50**	Delhi/CAS	OSDD518	India	AHHZ00000000	Contig
**51**		OSDD504		AHHY00000000	Contig
**52**		OSDD071		AHHX00000000	Contig
**53**	EAI-Manila	T92	San Francisco from a patient born in The Philippines	ABLN00000000	Scaffold
**54**		T46	USA (Born in Philippines)	ACHO00000000	Scaffold
**55**		T17		ABQH00000000	Scaffold
**56**		PR05	Malaysia	AOMG00000000	Contig
**57**		NA-A0008	India	ALYG00000000	Contig
**58**		NA-A0009	India	ALYH00000000	Contig
**59**		43-16836	Thailand	ATNF00000000	Contig
**60**	Haarlem	Haarlem	Netherlands	AASN00000000	Scaffold

**61**		str. Erdman = ATCC 35801	N/A	AP012340	Complete Genome
**62**		PanR0907	Panama	ATSO00000000	Chromosome
**63**		PanR0902		ATSK00000000	Chromosome
**64**		PanR0801		ATSG00000000	Chromosome
**65**		PanR0206		ATMN00000000	Chromosome
**66**		OSDD105	India	AUXD00000000	Contig
**67**		7199-99	Germany	HE663067	Complete Genome
**68**	F15/LAM4/KZN	KZN-1435	KwaZulu-Natal	CP001658	Complete Genome
**69**		KZN-V2475		ACVT00000000	Chromosome
**70**		KZN-R506		ACVU00000000	Chromosome
**71**		KZN 605		ABGN00000000	Scaffold
**72**		KZN-4207		CP001662	Complete Genome
**73**	LAM	UT205	Colombia	HE608151	Chromosome with gaps
**74**		MTB-476	Kazakhstan: Almaty	AZBA00000000	Contig
**75**		INS-XDR	Peru	JANH00000000	Contig
**76**		INS-SEN		JAQH00000000	Contig
**77**		INS-MDR		JAQI00000000	Contig
**78**		GM 1503	Gambia	ABQG00000000	Scaffold
**79**		98-R604 INH-RIF-EM	N/A	ABVM00000000	Scaffold
**80**		PanR0203	Panama	ATML00000000	Chromosome
**81**		PanR0602		ATRT00000000	Chromosome
**82**		PanR0402		ATEF00000000	Chromosome
**83**	LAM2	PanR0304	Panama	ATES00000000	Chromosome
**84**		PanR0503		ATRQ00000000	Chromosome
**85**		PanR0409		ATEA00000000	Chromosome
**86**		PanR0903		ATSL00000000	Chromosome
**87**		PanR0404		ATED00000000	Chromosome
**88**		PanR0308		ATEO00000000	Chromosome
**89**		PanR0313		ATEL00000000	Chromosome
**90**		PanR0610		ATSA00000000	Chromosome
**91**		PanR0201		ANZG00000000	Chromosome
**92**	LAM4	PanR1101	Panama	ATSS00000000	Chromosome

**93**		PanR1005		ANZI00000000	Chromosome
**94**		PanR0403		ATEE00000000	Chromosome
**95**	LAM1	PanR0411	Panama	ATDY00000000	Chromosome
**96**		PanR0804		ATSI00000000	Chromosome
**97**		PanR0407		ATEB00000000	Chromosome
**98**		PanR0412		ATDX00000000	Chromosome
**99**		PanR1006		ATST00000000	Chromosome
**100**		PanR0802		ANZH00000000	Chromosome
**101**		PanR0501		ATRP00000000	Chromosome
**102**		PanR0307		ATEP00000000	Chromosome
**103**		PanR0410		ATDZ00000000	Chromosome
**104**		PanR0317		ATEH00000000	Chromosome
**105**		PanR0311		ATEM00000000	Chromosome
**106**		PanR0904		ATSM00000000	Chromosome
**107**		PanR0803		ATSH00000000	Chromosome
**108**		PanR0609		ATRZ00000000	Chromosome
**109**		PanR0607		ATRY00000000	Chromosome
**110**		PanR0909		ATSQ00000000	Chromosome
**111**		PanR0601		ATRS00000000	Chromosome
**112**		PanR0314		ATEK00000000	Chromosome
**113**		PanR0207		ATMQ00000000	Chromosome
**114**		PanR0209		ATMP00000000	Chromosome
**115**		PanR0707		ATSE00000000	Chromosome
**116**		PanR0603		ATRU00000000	Chromosome
**117**		PanR0604		ATRV00000000	Chromosome
**118**		PanR0401		ATEG00000000	Chromosome
**119**		PanR0505		ATRR00000000	Chromosome
**120**		PanR0205		ATMM00000000	Chromosome
**121**		PanR0702		ATSC00000000	Chromosome
**122**	LAM3	PanR0906	Panama	ATSN00000000	Chromosome
**123**		PanR0805		ATSJ00000000	Chromosome
**124**		PanR1007		ATSR00000000	Chromosome
**125**		PanR0704		ANNN00000000	Chromosome
**126**		PanR0708		ATSF00000000	Chromosome

**127**		PanR0703		ATSD00000000	Chromosome
**128**		PanR0611		ATSB00000000	Chromosome
**129**		F11	Western Cape of South Africa	CP000717	Complete Genome
**130**	LAM9	MOS2	Russia	SRX216914	Reads
**131**	LAM9	MOS7	Russia	SRX216915	Reads
**132**	LAM9	SP34	Russia	SRX216908	Reads
**133**	LAM9	SP35	Russia	SRX216909	Reads
**134**	LAM9	SP36	Russia	SRX216910	Reads
**135**	LAM9	SP37	Russia	SRX216911	Reads
**136**	LAM9	SP38	Russia	SRX216912	Reads
**137**	LAM9	SP39	Russia	SRX216913	Reads
**138**	LAM9	CTRI-2	Russia	CP001662	Complete Genome
**139**	SMI-049	S96-129	Sweden	AEGB00000000	Chromosome
**140**	SMI-049	BTB05-559	Sweden	AEGD00000000	Chromosome
**141**	SMI-049	BTB05-552	Sweden	AEGC00000000	Chromosome
**142**	S	SUMu002	N/A	ADHR00000000	Scaffold
**143**	S	SUMu003	N/A	ADHS00000000	Scaffold
**144**	S	SUMu004	N/A	ADHT00000000	Scaffold
**145**	S	SUMu005	N/A	ADHU00000000	Scaffold
**146**	S	SUMu006	N/A	ADHV00000000	Scaffold
**147**	S	SUMu008	N/A	ADHX00000000	Scaffold
**148**	S	SUMu007	N/A	ADHW00000000	Scaffold
**149**	S	SUMu009	N/A	ADHY00000000	Scaffold
**150**	S	GuangZ0019	China	ANFI00000000	Scaffold
**151**	X	CDC1551	USA	AE000516	Complete Genome
**152**	X	CDC1551A	USA	AELF00000000	Scaffold
**153**	X	PanR0316	Panama	ATEI00000000	Chromosome
**154**	X	PanR0301	Panama	ATET00000000	Chromosome
**155**	X	PanR0305	Panama	ATER00000000	Chromosome
**156**	Ural	MOS9	Russia	SRX216916	Reads
**157**	Ural	SP24	Russia	SRX216901	Reads
**158**	Ural	SP25	Russia	SRX216902	Reads

**159**		SP28		SRX216903	
**160**		SP31		SRX216905	
**161**		SP32		SRX216906	
**162**		SP33		SRX216907	
**163**		OSDD493	India	AVQJ00000000	Contig
**164**	T	PanR0309	Panama	ATEN00000000	Chromosome
**165**		PanR0208		ATMO00000000	Chromosome
**166**		PanR0202		ATAS00000000	Chromosome
**167**		PanR0908		ATSP00000000	Chromosome
**168**		H37Rv	N/A	CP003248	Complete Genome
**169**		H37Ra	N/A	CP000611	Complete Genome
**170**	Unknown	SUMu012	N/A	ADIB00000000	Scaffold
**171**		SUMu010		ADHZ00000000	Scaffold
**172**		SUMu11		ADIA00000000	Scaffold
**173**		SUMu01		ADHQ00000000	Scaffold
